# 661. Phase 1, dose-escalation trial of the safety and pharmacokinetics of SARS-CoV-2 DNA-encoded monoclonal antibodies (DMAb) in healthy adults

**DOI:** 10.1093/ofid/ofaf695.216

**Published:** 2026-01-11

**Authors:** Pablo Tebas, Ami Patel, Joseph Agnes, Elizabeth M Parzych, Amanda Baer, Maria Caturla, Sukanya Ghosh, Mansi Purwar, Nicole Bedanova, chungdhak Tsang, Knashawn Morales, Dinah Amante, Paul D Fisher, Joe Francica, Laurent Humeau, Jesper Pallesen, Paul Eric Leon, Mark T Esser, Trevor R F Smith, David B Weiner

**Affiliations:** University of Pennsylvania, Merion Station, PA; Wistar Institute, Philadelphia, Pennsylvania; INOVIO Pharmaceuticals, Plymouth Meeting, Pennsylvania; Wistar Institute, Philadelphia, Pennsylvania; University of Pennsylvania, Merion Station, PA; University of Pennsylvania, Merion Station, PA; Wistar Institute, Philadelphia, Pennsylvania; Wistar Institute, Philadelphia, Pennsylvania; Wistar Institute, Philadelphia, Pennsylvania; University of Pennsylvania, Merion Station, PA; Wistar Institute, Philadelphia, Pennsylvania; INOVIO Pharmaceuticals, Plymouth Meeting, Pennsylvania; Inovio Pharmaceuticals, Plymouth Meeting, Pennsylvania; AstraZeneca, Gaithersburg, Maryland; INOVIO Pharmaceuticals, Plymouth Meeting, Pennsylvania; Wistar Institute, Philadelphia, Pennsylvania; AstraZeneca, Gaithersburg, Maryland; AstraZeneca, Gaithersburg, Maryland; Inovio Pharmaceuticals, Plymouth Meeting, Pennsylvania; Wistar Institute, Philadelphia, Pennsylvania

## Abstract

**Background:**

Local intramuscular administration of synthetic plasmid DNA (pDNA) encoding monoclonal antibodies (mAbs) offers a promising alternative to traditional recombinant protein-based mAb delivery. This approach may enable durable in vivo expression of functional antibodies and overcome limitations related to cost, production, and cold-chain logistics. AZD5396 and AZD8076 are modified versions of the SARS-CoV-2 neutralizing antibody cocktail Evusheld, encoded as DNA-delivered monoclonal antibodies (DMAbs).

**Figure:**
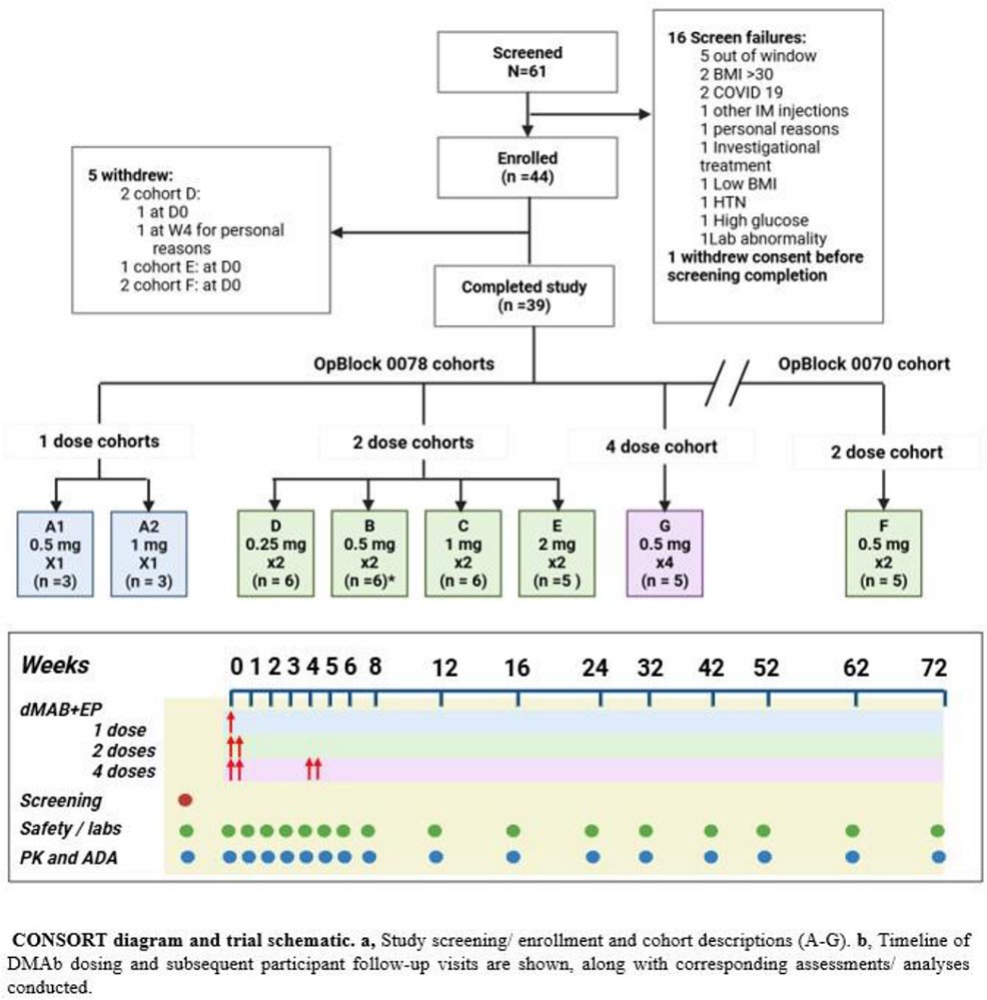
CONSORT diagram and trial schematic Longitudinal serum concentration of in vivo-expressed DMAbs AZD5396 and AZD8076

**Figure:**
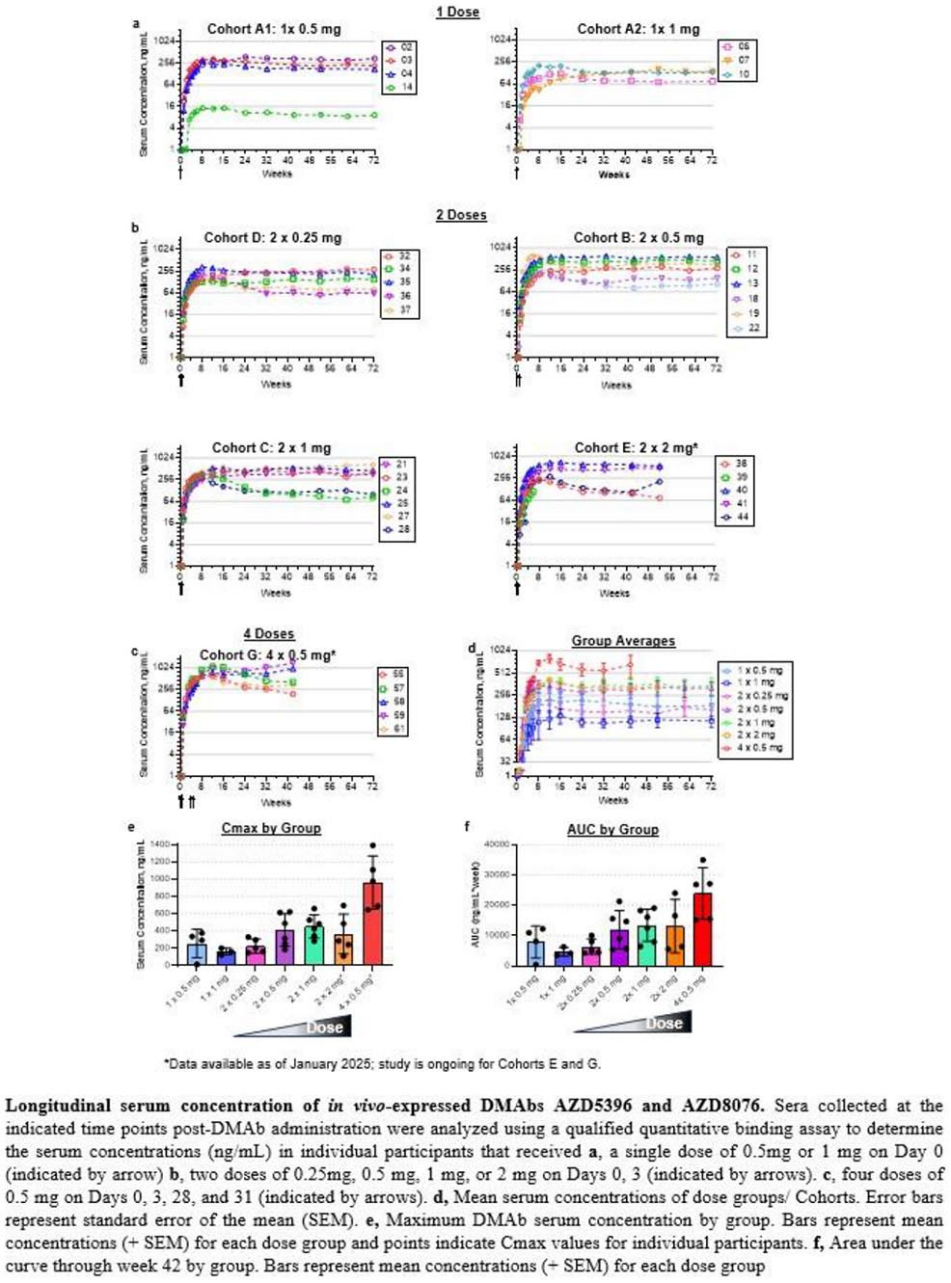

**Methods:**

In this Phase 1, dose-escalation study (ClinicalTrials.gov identifier: NCT05293249), we evaluated the safety, tolerability, and pharmacokinetics of a pDNA cocktail encoding AZD5396 and AZD8076 in healthy adults. Participants received up to four intramuscular doses of the pDNA cocktail delivered by CELLECTRA™ electroporation. The primary endpoints were safety and pharmacokinetics. Exploratory endpoints included anti-drug antibody (ADA) development and functional activity against SARS-CoV-2 variants.

**Results:**

All 44 enrolled participants received at least one dose, and DMAbs were detected in 100% of evaluable participants (n=39). Serum DMAb concentrations reached a mean peak of 1.11 µg/mL, with sustained expression observed in all participants who completed 72 weeks of follow-up. The product was well tolerated, and no product-related serious adverse events were reported. Exploratory analyses demonstrated binding to multiple SARS-CoV-2 spike variants and neutralizing activity in pseudovirus assays. Across ∼1,000 serum samples, no ADAs were detected using validated tiered assays.

**Conclusion:**

These findings provide the first-in-human proof-of-concept that synthetic pDNA DMAb technology enables durable in vivo production of a functional mAb cocktail. The results highlight the critical role of optimized synthetic design, formulation, and delivery in achieving biologically relevant expression. DNA-delivered mAbs may represent a long-acting, scalable, cold-chain-independent platform for targeting a wide range of diseases treatable with antibody-based therapeutics.

**Disclosures:**

All Authors: No reported disclosures

